# Quartet decomposition server: a platform for analyzing phylogenetic trees

**DOI:** 10.1186/1471-2105-13-123

**Published:** 2012-06-07

**Authors:** Fenglou Mao, David Williams, Olga Zhaxybayeva, Maria Poptsova, Pascal Lapierre, J Peter Gogarten, Ying Xu

**Affiliations:** 1 Department of Biochemistry and Molecular Biology, University of Georgia, 120 Green St, Athens, GA, 30622, USA; 2 Department of Molecular and Cell Biology, University of Connecticut, 91 North Eagleville Road, Storrs, CT, 06269, USA; 3 Biotechnology-Bioservices Center, University of Connecticut, Storrs, CT, 06269-3149, USA; 4 Department of Biology, West Virginia University, 53 Campus Drive, Morgantown, WV, 26506-6057, USA; 5 College of Computer Science and Technology, Jilin University, Changchun, Jilin, China; 6 Present Address: Department of Biological Sciences, Dartmouth College, 78 College Street, Hanover, NH, 03755, USA

## Abstract

**Background:**

The frequent exchange of genetic material among prokaryotes means that extracting a majority or plurality phylogenetic signal from many gene families, and the identification of gene families that are in significant conflict with the plurality signal is a frequent task in comparative genomics, and especially in phylogenomic analyses. Decomposition of gene trees into embedded quartets (unrooted trees each with four taxa) is a convenient and statistically powerful technique to address this challenging problem. This approach was shown to be useful in several studies of completely sequenced microbial genomes.

**Results:**

We present here a web server that takes a collection of gene phylogenies, decomposes them into quartets, generates a Quartet Spectrum, and draws a split network. Users are also provided with various data download options for further analyses. Each gene phylogeny is to be represented by an assessment of phylogenetic information content, such as sets of trees reconstructed from bootstrap replicates or sampled from a posterior distribution. The Quartet Decomposition server is accessible at http://quartets.uga.edu.

**Conclusions:**

The Quartet Decomposition server presented here provides a convenient means to perform Quartet Decomposition analyses and will empower users to find statistically supported phylogenetic conflicts.

## Background

Sequence data revealed that genetic material in prokaryotes (bacteria and archaea) can be transferred between divergent organisms [1] to an extent that makes it difficult to reconstruct their evolutionary history [2-4]. Many microorganisms can take DNA directly from the environment; phages infect prokaryotic cells and may bring new DNA fragments into the host genomes; the conjugation machinery allows for DNA exchange directly between cells; and phage derived gene transfer agents [5] were suggested to transfer genetic material between related and possibly unrelated organisms [6]. Gene transfer results in genes found in the same genome to have different phylogenies. The currently popular strategies for inference of organismal relationships include (i) construction of an organismal tree based on conserved genes presumed to be not transferred such as 16S ribosomal RNA and ribosomal proteins, or (ii) the assumption that the plurality phylogenetic signal contained in all genes reflects the organismal history. The plurality signal is either extracted through joint analysis of several genes, usually after removing genes that show signs of having been horizontally transferred [7], or individual gene trees are combined using a variety of supertree approaches [8,9].

Phylogeny is typically represented as a tree, often with tens or hundreds of leaves. The large size and unequal number of taxa makes comparisons between trees difficult. A common approach is to compare all significantly supported bipartitions. Lento plots allow visualizing the bipartitions supported by many gene families, and also depict, for each bipartition, all those bipartitions that are in conflict [10-12]. As well as requiring all phylogenies to be the same size *i.e.*, all gene families represented in all genomes analyzed, bipartition-based approaches suffer from a loss of resolution as more sequences and therefore tips and edges are included. Quartet Decomposition avoids both of these problems [13,14].

**Figure 1:**
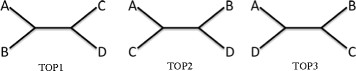
**Quartet topologies.** The three possible quartet topologies for four taxa **A**, **B**, **C** and **D**.

Quartets are unrooted trees consisting of four taxa (Figure 1). A quartet is the minimal informative unit in a tree, and it has three possible topologies. An unrooted three-taxon tree unit only has one topology and thus is not informative, while a five-taxon tree unit has fifteen topologies, thus is too complicated; the four-taxon tree unit has a good balance between the amount of information it can carry and the complexity involved in analyzing it [15]. Quartet Decomposition is the analysis of quartets embedded in larger phylogenies.

**Figure 2:**
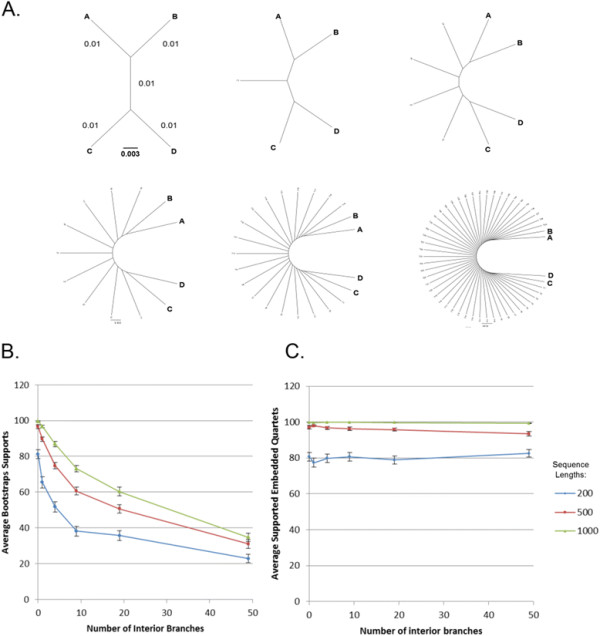
**Comparison of the performance of bipartiton and quartet-based analyses.** Increasing taxon sampling justifiably is expected to increase the reliability of phylogenetic reconstruction; however, the support values for bipartitions that include all taxa tends to drop as more taxa are added. Panel **A** depicts the phylogenies used for simulations. Starting with an unrooted tree of four leaves, ((**AB**),(**CD**)) and an internal branch of 0.01 average substitutions per site, we added 1, 4, 9, 19 and 49 additional leaves to the internal branch. Simulations for each topology were performed with Seq-Gen. [16] using the indicated trees, the WAG substitution matrix [17] and a Γ distribution with a shape parameter of 1 approximated by four discrete rate categories for the rate distribution. SEQBOOT from the PHYLIP package [18] was used to generate 100 bootstrap sequences and trees were reconstructed from each bootstrap sample using FastTree 2.1 [19] using the same model for sequence evolution and parameters “-spr 4”, “-mlacc 2”, and “-slownni” for increased reconstruction accuracy. For each topology the evolution of sequences of varying lengths (200, 500 and 1000 amino acids) was simulated. For each of the simulated data sets, we generated 100 bootstrap replicates and recorded the maximum support for a bipartition separating (**AB**) from (**CD**) (Panel **B**) and the bootstrap support for the embedded quartet ((**AB**),(**CD**)) for all simulations (Panel **C**). Error bars give the standard error of the mean from 100 replicates each

Support for bipartitions that include all taxa present in a phylogenetic tree can decrease, if one sequence in a larger phylogeny has low phylogenetic signal causing its position among bootstrap replicates to vary. In addition, as more taxa are added to an analysis, the shorter the internal branches, and the lower their support values become. This situation is unsatisfactory, because increased taxon sampling is expected to increase the reliability of the phylogenetic reconstruction; however, the increase in reliability is not reflected in increased bipartition support values. To illustrate this paradox we performed simulations summarized in Figure 2. Figure 2A shows how the simulation is performed: starting from a tree with four tips, we grow the tree by adding more tips at the internal branch; and then generate replicates, carry out bipartition and quartet-based analysis. Figure 2B shows that even for sequences 1000 amino acids long, with 10 additional tips, the maximum support for a bipartition separating AB from CD is less than 80% on average, and with 20 additional tips it is close to 60%, too low to provide insight into any biological processes. In contrast, Figure 2C shows the ((A,B),(C,D)) embedded quartet is present in almost all replicates, demonstrating the near independence of sample size and embedded quartet resolution.

The use of quartets has been explored in various phylogenetic applications. In 1996 K. Strimmer and A. von Haeseler developed the quartet puzzling algorithm for tree reconstruction [20]. Since then a quartet-based software TREE-PUZZLE [21] has been developed and widely used for tree reconstruction from DNA and protein sequences. Later, two software packages, Clann [22] and QuartetSuite [23], were developed allowing construction of supertrees from multiple trees using quartets. Zhaxybayeva and Gogarten [24] introduced the use of embedded quartets to solve the taxon-sampling problem usually associated with quartet based analyses [25], and used the analysis of embedded Quartet Decomposition to examine gene histories in cyanobacteria, and to identify horizontally transferred genes [13,14]. Boc *et al.* recently developed a Horizontal Gene Transfer (HGT) detection algorithm that uses a quartet-based distance as one of the criteria when reconciling gene and organismal phylogenies [26]. Quartet analysis is also a good choice for multi-locus sequence data analysis [27], and has been used to infer taxonomic relationships [28,29] as well as tree-like and net-like evolutionary processes [30].

To facilitate a wider application of Quartet Decomposition, we present a web-based platform for decomposing a given set of trees into quartets. The web server also provides several quartet-based analysis tools such as quartet spectrum generation, agreement score calculation, and split network generation. Considering that a user may want to carry out additional analyses of the quartets, we also provide several options to download the computed quartets.

Given a gene tree, our algorithm enumerates all possible combinations *M* of any four out of *x* total taxa under consideration,

(1) $$M=\left(\begin{array}{c} x\\ 4 \end{array}\right)$$

Let’s use A, B, C and D to represent the four taxa in a specific embedded quartet of the full phylogenetic tree. In order to determine what specific topology the embedded quartet has, we calculate pairwise distances *d*_AB_, *d*_AC_, *d*_AD_, *d*_BC_, *d*_BD_ and *d*_CD_, where the distance *d*_XY_ is defined as the sum of all branch lengths in the given tree from leaf X to leaf Y. If (*d*_AC_ + *d*_BD_)-(*d*_AB_ + *d*_CD_) > 0, the quartet has topology TOP1 (Figure 1); if (*d*_AD_ + *d*_BC_)-(*d*_AC_ + *d*_BD_) > 0 - topology TOP2 ; and if (*d*_AB_ + *d*_CD_)-(*d*_AD_ + *d*_BC_) > 0 - topology TOP3. Each branch of the embedded quartet may correspond to several internal edges of the full phylogeny and has a length calculated as exemplified for topology TOP1 (Figure 1): the length of the internal branch is *d*_*internal*_ = [(*d*_AC_ + *d*_BD_)-(*d*_AB_ + *d*_CD_)]/2, and the length of the external branch of taxon A is *d*_*A*_ = [(*d*_AC_ + *d*_AD_)- *d*_CD_]/2- *d*_*internal*_. The lengths of other external branches are calculated similarly.

## Implementation

The server is implemented on a computer running Linux RedHat Enterprise 5.0 operating system. Apache 2.2.9 is used as the web server, and PHP 5.2.6 is used to develop dynamic webpages. Scripts implementing the server functions are written in Perl. The BioPerl 1.60 [31] TreeIO module is used to help compute the decomposition of an input tree, and the Perl graphic library GD is used to draw the quartet spectrum. SplitTree4 [32] is used to generate the split network. A Linux computer cluster with 8 nodes which can support 32 simultaneous jobs is used as the backend for tree decomposition calculation. The Sun Grid Engine 6.2 is used for job management.

**Figure 3:**
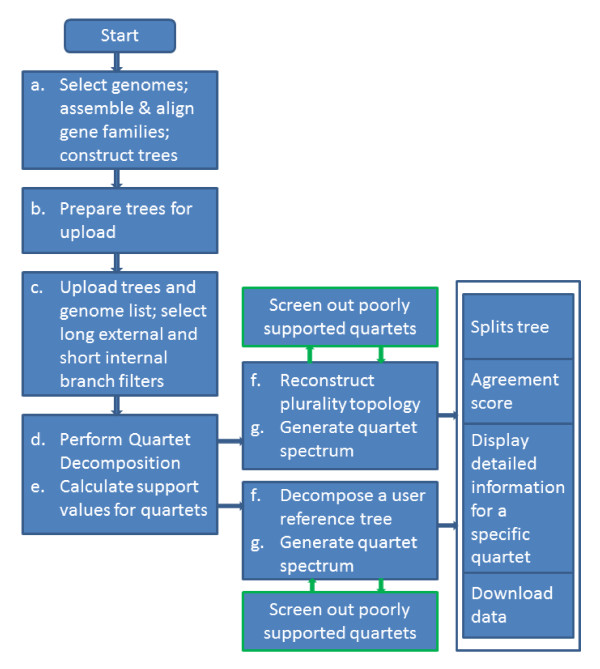
**Data flow of quartet decomposition analyses using the QD server.** Steps labeled a-g are described in detail in the Results and Discussion section. The boxes outlined with green border are parameterized filters, which can be applied multiple times to generate different quartet spectra. The green arrows represent the repeatable steps.

The overall structure of the server is illustrated in Figure 3. A user needs to prepare two input files: one containing the names of the genomes or taxa under consideration, the other is a compressed file of all gene trees (currently the server will accept .tar.gz, .rar and .zip files). Each gene tree is represented by multiple trees that assess phylogenetic information content, such as sets of trees reconstructed from bootstrap replicates or sampled from a posterior distribution. We also provide an interface for users to generate bootstrap replicates from multiple sequence alignment. The replicates are generated by a BioPerl utility function, and the trees are generated by FastTree 2.1 [19]. Since we are comparing quartets across gene families to obtain a plurality signal, taxa labels corresponding to genes in the same organism are expected to have the same name. To facilitate the replacement of gene identifiers with the names of the genomes, we provide Perl scripts (see FAQ section in the server) for conversion and consistency checks. These scripts require BioPerl 1.60 or newer version on the user’s computer. If the user does not have BioPerl installed in their local computer, we also provide a web interface for the user to do the name conversion in the server.

After the name conversion, the user can upload the files to the server, specify the parameter values (or just use the default parameter values given by the server), and start the decomposition calculation. The computation may take several hours depending on the number of taxa, the number of gene families and the number of trees per gene family. For example, when we provided trees from 100 bootstrap samples for each gene family, it took 2 hours and 10 minutes for a job with 1128 gene families from 10 genomes, and 15 hours and 21 minutes for a job with 1734 gene families from 19 genomes. The run time is heavily dependent on the number of genomes since the number of quartets is a fourth degree polynomial of this number. Due to the limitations of computer hardware housing the server at the time of writing (May 2012), we suggest the user not to submit a job with more than 20 genomes. However, the server will accept a job with up to 100 genomes, issuing a warning for a job with more than 40 genomes. The user can refresh the job status page while the job is running: the server will display the currently analyzed gene family. The server will send an email to the user with a link to the status page once the job is submitted; and it will send another email after the job is completed. After the decomposition is done, a quartet spectrum [14] (see next section for its description) will be generated, and the user can run various analyses using tools provided by server, such as filtering quartets, calculating an agreement score, downloading a specified subset of the decomposed quartets, and generating a splits network.

## Results and discussion

The server provides a platform for performing the following quartet-based analyses.

### Quartet spectrum generation

**Figure 4:**
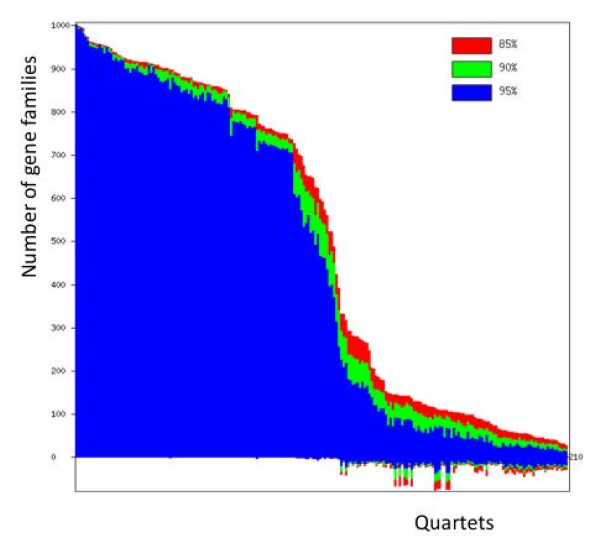
**An example of a quartet spectrum.** The x-axis represents the quartets, one per column, arranged in descending order of the number of gene families supporting the plurality/reference topology of each quartet. For each column, the y-axis represents the number of gene families in which that quartet supports (positive y values, one topology) or conflicts (negative y values, the other two possible topologies) with the plurality or reference topology. For the conflicts, the y value represents a sum of gene families supporting the two other topologies. The spectrum is color-coded according to different bootstrap support thresholds used.

Quartet Decomposition of a gene tree is the process of finding all possible embedded quartet topologies for a given tree. For a given list of genomes and multiple gene families collected from these genomes, the quartet topologies in a specific gene family are identified, and for the set of taxa summarized in a quartet spectrum. The calculation consists of the following steps (the user needs to perform steps a-c, the server performs steps d-g):

a. For a set of genomes of interest, assemble and align gene families, and obtain trees either from bootstrap replicates or from a posterior distribution.

b. Prepare trees in Newick format for each gene family. Put all trees for the same gene family to one file. Compress all tree files to a single file.

c. Upload genome list and the compressed tree file to the server. Specify the parameters for filters (see below). Start the job.

d. Decompose each tree into embedded quartets.

e. For each gene family, calculate the support value for the three topologies of each quartet by counting the fraction of the bootstrap trees that contain this quartet topology. In case of 100 replicate trees, each embedded quartet in a family has a dominant topology with a maximum score of 100. Comparable scores for the alternative quartet topologies, such as 34, 33, 33, are indicative of no or little phylogenetic signal for that embedded quartet in a particular gene phylogeny.

f. For each quartet, determine the plurality topology across all gene families as follows: given a threshold for a support value cut-off to determine whether the dominant topology is supported (85%, 90% and 95% are currently supported by the server), count the number of gene families supporting each of the three topologies. The topology with the highest number of supporting gene families is considered the plurality topology of the quartet among all the analyzed gene families.

g. Sort the quartets by the number of gene families supporting the plurality topologies, and plot as a histogram with these sorted numbers along with the labels of the associated quartets. Analogous to the Lento plot [10], another histogram on the negative side of the Y-axis is also added to show the sum of the two non-plurality topologies (conflicting topologies) for each quartet. The resulting diagram is called the quartet spectrum (Figure 4).

The quartet spectrum provided by the server is interactive: when a user clicks on the bar representing a specific quartet, a new page pops-up with the detailed information for that quartet, including its support value in each gene family.

Sometimes a user may prefer to compare the individual gene phylogenies against another tree obtained from other sources, such as phylogenies calculated from ribosomal components [33], the Tree of Life Project (http://tolweb.org/tree/), or the NCBI taxonomy database [34] (http://www.ncbi.nlm.nih.gov/Taxonomy/Browser/wwwtax.cgi). The server can compare the quartets in the gene families against the quartet topologies embedded in the reference tree and generate a quartet spectrum counting the quartet topologies in the reference tree as positive. Large values in the negative part of the spectrum would indicate specific conflicts between gene phylogenies and the reference tree. The presence of at least one embedded quartet with a bootstrap support value greater than 80 in conflict with a reference phylogeny reveals a significant phylogenetic conflict suggestive of an HGT event. Depending on the data analyzed, alternative explanations for phylogenetic conflict may need to be considered. Lineage sorting occurs in taxa with large populations and a rapid succession of speciation events; unrecognized paralogy always is an alternative explanation to HGT [35] and needs to be considered when independent and parallel gene loss cannot be excluded because only few lineages are analyzed. While the rate of false positives is reasonably assessed through the bootstrap support values [14,36], the rate of false negatives likely is large, especially for transfers between close relatives [37].

### Processing of paralogs

If there are paralogs in a gene family (and hence multiple homologs per gene family have the same label), the distribution of quartet topologies will be calculated as follows. Given a tree and four genomes A, B, C and D, the number of paralogs are *a*, *b*, *c* and *d* for each genome respectively. The total number of quartet topologies with the four genomes will be *t* = *a* × *b* × *c* × *d*. Since each topology will represent one of TOP1, TOP2 or TOP3 (see Figure 1), we can count the total number of quartet topologies with TOP1, TOP2 and TOP3 as *t*_*1*_, *t*_*2*_ and *t*_*3*_. The sum of *t*_*1*_, *t*_*2*_ and *t*_*3*_ is equal to *t*. For the given tree, we calculate the ratio of TOP1, TOP2 and TOP3 as *t*_*1*_/*t*, *t*_*2*_/*t* and *t*_*3*_/*t*, respectively. The sum of the three ratios will be equal to 1, which is the same for a tree without paralogs. In addition, quartets with two tips from same genome (*i.e.*, paralogs) will be ignored. If gene families with paralogs are included in a quartet decomposition analysis, conflicting quartets may reflect the gene duplication events, and can no longer be identified with gene transfer events. However, families with paralogs are useful to extract the plurality phylogenetic signal contained in a set of genomes.

### Agreement score calculation

For each gene family we also calculate an *agreement score*[13], which measures how well the gene family agrees with the plurality or the reference tree:

(2) $$\text{S=}\frac{\sum_{i=1}^{M}n_{i}}{\text{N}*\text{M}}$$

where *N* is the number of trees for this gene family; *M* is the number of possible quartets; and *n*_*i*_ is the number of topologies that agree with the plurality (or reference) for the *i*^*th*^ quartet. The score *S* is equal to 1 if all the trees have the same topology which is also identical to the reference, and it is less than 1 otherwise. The more conflicts between the gene trees and the reference are observed, the closer the score is to 0.

### Filters

The inaccuracies in phylogenetic reconstruction may introduce noise and misleading information to quartet analysis. To minimize their impact, we designed three filters to remove such quartets, categorized as follows.

#### Long external branch(es)

Each quartet has four external branches and one internal branch (Figure 1). Long external branches may lead to the so called *long branch attraction* artifact [38], which may erroneously lead to the conclusion that two rapidly evolving lineages are closely related. A filter is implemented to remove quartets with long external branches according to the following criterion: if the ratio between the longest external branch and the internal branch is larger than a pre-set threshold (default value is 10), it will be removed.

#### Short internal branch

If a quartet has a very short internal branch, there may not be enough phylogenetic information to resolve the topology correctly. The server provides an option to remove a quartet if its internal branch is shorter than a pre-set threshold (default value is 0.02 substitutions per site). If the branch length in the tree is not measured by substitutions per site, 0.02 may not be an appropriate value, and the user has to decide a proper value by himself.

#### Less supported quartets

Quartets that due to a lack of phylogenetic signal are poorly resolved in most gene families could result in erroneous but significant conflicts with the plurality (false positives) [14]. To remove quartets that are not resolved by most gene families, we implemented the following filter, defined by two thresholds, *T*_*1*_ (ranges between 0% and 100%) and *T*_*2*_ (a positive integer). For a specific quartet, if the proportion of the gene families supporting it with a support value of at least *T*_*1*_ is less than *T*_*2*_, this quartet will be removed from a quartet spectrum. This filter is applied after the decomposition process is done, and the effect of different filter settings on the quartet spectrum can be explored. In contrast, the other two filters have to be specified before the decomposition process starts.

### Splits network generation

A *splits network* is a network representation of the relationship of a set of taxa [39], in which multiple alternative splits (and not just the most supported one) are depicted. In situations with frequent exchanges of genetic material, a split network is a better representation for the taxa relationship than a tree. Our server can convert any quartet subset (see next section for a description of quartet sets) to a matrix [40,41], and then generate a split network by using the SplitTree4 program [32].

### Quartet download

Although we have provided a number of quartet analysis tools through the server, a user may want to perform his/her own analyses on the computed quartets. We offer two options to download the decomposed quartets.

The first option is to download a subset of the quartets that are supported with a support value of at least *T*_*1*_ in at least *T*_*2*_ gene families (see section on filters for descriptions of *T*_*1*_ and *T*_*2*_). The second option is based on the quartet spectrum. The quartet topologies in agreement with the plurality are considered as plurality quartet topologies, and as conflicting quartet topologies otherwise. The user can obtain the subsets of plurality or conflicting quartet topologies using thresholds *T*_*1*_ and *T*_*2*_ as described above.

### Examples

We provide two quartet decomposition examples, which can be accessed from the Frequently Asked Questions section on the quartet server web page. Both the data sets and the quartet spectrum are available on the server. The user can run the job by using the data sets, or go directly to the quartet spectrum and explore other analyses on the server.

One data set consists of 1,128 gene families present in at least 9 of 11 selected cyanobacterial genomes [14]. Quartet Decomposition of these families revealed that cyanobacterial evolution is incompatible with strictly bifurcating tree and helped to pinpoint specific cases of horizontal gene transfer.

The other data set consists of 1,812 gene families present in at least 4 of 18 specific cyanobacterial genomes of *Prochlorococcus marinus* and marine *Synechococcus* spp. [13]. Quartet Decomposition identified 495 gene families that did not separate genera *Prochlorococcus* and *Synechococcus* as expected. This observation can be explained by the existence of a “highway of gene sharing” between marine *Synechococcus* spp. and low-light adapted *Prochlorococcus* spp. (see [13] for additional discussion).

In both studies the Quartet Decomposition has proven to be an invaluable tool for identification of phylogenetic signal shared by genes in analyzed genomes and for discovery of horizontally transferred genes.

## Conclusion

The Quartet Decomposition server presented here provides an interactive interface to dissect complex evolutionary histories of microbial genomes. We believe that this online service will be a valuable tool for the comparative genomics community.

## Availability and requirements

Project name: Quartet Decomposition server.

Project home page: http://quartets.uga.edu.

Operating system(s): Platform independent

Other requirements: The server has been tested using Firefox (Windows, Linux and Mac OS X), Internet Explorer (Windows), Safari (MacOS X Lion), and Google Chrome (Windows and Linux) browsers.

## Competing interests

The authors declare that they have no competing interests.

## Authors’ contributions

FM implemented the server and drafted the paper, DW, MP and OZ tested the server, PL performed the simulation study about bipartition and quartet-based comparison study, OZ contributed the example data, JPG conceived and together with YX supervised the project. All authors contributed to the writing of the manuscript.
